# The addicted brain: understanding the neurophysiological mechanisms of addictive disorders

**DOI:** 10.3389/fnint.2015.00018

**Published:** 2015-03-19

**Authors:** Melissa A. Herman, Marisa Roberto

**Affiliations:** Committee on the Neurobiology of Addictive Disorders, The Scripps Research InstituteLa Jolla, CA, USA

**Keywords:** alcohol, GABA, cocaine, endocannabinoid, sex-differences, opioids, nicotine

The consumption of chemical substances that produce transient feelings of euphoria or pleasure and the development of dependence on those substances by a subset of individuals is as old as the human race itself. Currently, the cost of addiction to illicit drugs in the United States is more than 600 billion dollars a year (National Institute on Drug Abuse, 2015), with profound social and economic impacts. Despite the prevalence and long history of addiction, it is still not clear what neurophysiological processes are involved in the development and progression of addictive disorders. The challenge of current and future studies is to understand how alcohol and drugs alter specific brain systems to influence tolerance and/or lead to the addicted state with the overarching goal of identifying vulnerable populations and improving on current treatment strategies.

Drug addiction is defined as a chronic relapsing disorder that is comprised of three stages: preoccupation/anticipation, binge/intoxication, and withdrawal/negative affect. These three stages are conceptualized as feeding into one other, becoming more intense over time, and ultimately leading to the pathological state known as addiction. Different drugs produce distinct patterns of addiction that engage different components of the addiction cycle, depending on dose and length of use. As an individual moves from being a “user” to “abuser” and then to “addicted” a shift occurs from positive reinforcement driving the motivated behavior to negative reinforcement driving the motivated behavior. Importantly, the progression of drug addiction involves alterations in normal brain circuitry that result in long-lasting drug-induced neuroplastic changes (Koob and Volkow, 2010). Critical neurotransmitters (i.e., gamma-aminobutyric acid, glutamate, dopamine, opioid peptides, serotonin, acetylcholine, endocannabinoids, corticotropin releasing factor) and neurocircuits (i.e., ventral tegmental area, nucleus accumbens, amygdala, cerebellum, prefrontal cortex) underlie the pathological changes at each of these stages (Figure 1). A better understanding of the main cellular mechanisms and circuits affected by chronic drug use and the influence of environmental stressors, developmental trajectories, and genetic factors on these mechanisms will lead to a better understanding of the addictive process and to more effective therapeutic strategies for the prevention and treatment of substance-use disorders. In the special topic Frontiers journal “the neurobiology of addictive disorders,” we provide important breakthroughs on the actions of commonly abused addictive substances (i.e., alcohol, cocaine, nicotine, cannabinoids) on the function of neuronal circuits.

**Figure 1:**
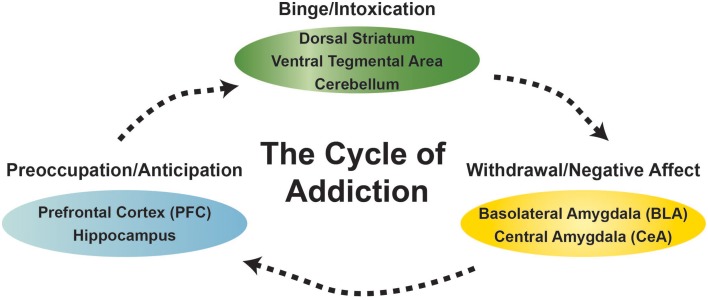
**Diagram of the behavioral states and select brain areas discussed in this research topic associated with the cycle of addiction**.

Alcohol and drugs of abuse represent unique experimental challenges as they often engage multiple molecular and intracellular systems in distinct brain regions. Current work seeks to identify these molecular targets and how they are altered by acute and/or chronic exposure. One important molecular target is phosphodiesterase 10A (*Pde10a*), a regulator of cyclic nucleotide activity. Logrip and Zorrilla (2014) found that expression of *Pde10a*, which is associated with relapse-like ethanol self-administration, was differentially altered during different stages of alcohol withdrawal in the rat. During acute withdrawal *Pde10a* expression was increased in the basolateral amygdala and medial prefrontal cortex. During protracted withdrawal *Pde10A* expression remained elevated in contrast to what has been observed in other brain regions.

Another emerging target is extracellular signal-regulated kinase (ERK). Zamora-Martinez and Edwards (2014) reviewed emerging data in the important role of ERK activity in the brain on the development and progression of drug and alcohol addiction. Varodayan and Harrison (2013) investigated the molecular mechanisms underlying alcohol's effects on neurotransmitter release at the presynaptic terminal. This study indicated that alcohol induces heat shock factor 1 transcriptional activity to trigger a specific coordinated adaptation in GABAergic presynaptic terminals in cultured cortical neurons. This mechanism could explain some of the changes in synaptic function that occur soon after alcohol exposure, and may underlie some of the more enduring effects of chronic alcohol intake on local circuit function.

Addiction engages many brain regions at different stages of the development of the disorder. Ongoing studies target distinct brain regions to pinpoint the specific intracellular pathways employed by alcohol and drugs of abuse in the development of dependence. Nimitvilai et al. (2013) found that ethanol-induced excitation of dopamine neurons in the rat ventral tegmental area (VTA) was significantly reduced in the presence of a phorbol ester in a mechanism involving the theta isoform of protein kinase C. These results shed new light on how ethanol alters the activity of the reward pathway, specifically the activity of dopamine neurons that mediate the salience of “pleasurable” stimuli. Soares-Simi et al. (2013) investigated changes in cyclic adenosine monophosphate response element-binding protein (CREB) DNA-binding activity in the prefrontal cortex and hippocampus of adolescent and adult mice in the context of alcohol-induced behavioral sensitization. Significant and differential neuroadaptive changes in CREB DNA-binding activity were reported in adolescent mice compared with adult mice. These differences may contribute to the blunted ethanol-induced behavioral sensitization observed in adolescent mice.

In addition to engaging molecular signaling pathways, alcohol and drugs of abuse also produce changes in ion channels to alter neuronal activity. For example, Kreifeldt et al. (2014) assessed the role of specific large conductance calcium-activated potassium (BK) channel subunits in voluntary ethanol consumption and found that the selective deletion of the β1 or β4 auxiliary subunit did not influence consumption in nondependent mice but did produce opposite effects on drinking during withdrawal from chronic intermittent ethanol. The results of this study suggest that auxiliary subunits of BK channels may represent a novel therapeutic target for the treatment of alcoholism. In another study, Botta et al. (2014) examined the effect of ethanol on the brief suppression of firing of cerebellar Golgi cells induced by stimulation of granule cell axons in the rat cerebellum. Acute ethanol diminished the pause in Golgi cell firing, an effect that was mimicked by partial inhibition of the Na^+^/K^+^ pump (ATPase). This reduction in feedback inhibition represents one way in which ethanol can dysregulate cerebellar function that may contribute to alcohol intoxication. Alcohol has also been shown to engage the immune pathway. Gruol et al. (2014) used a transgenic mouse model overexpressing the immune cytokine CCL2 to determine if elevated levels of CCL2 interact with the effects of ethanol in the hippocampus and found that elevated levels of CCL2 protected against the effects of acute ethanol on synaptic plasticity (i.e., LTP).

Alcohol and drugs of abuse interact with many peptide and neurotransmitter systems in distinct regions of the brain. Bajo et al. (2014), investigated the effects of morphine on GABAergic transmission in rat central amygdala (CeA) neurons and found that acute and chronic morphine exposure and withdrawal alters opioid and GABA signaling in the central amygdala. These findings suggest that during the acute phase of withdrawal, the CeA opioid and GABAergic systems undergo neuroadaptative changes conditioned by a previous chronic morphine exposure and dependence. McCool et al. (2014) found that the activation of 5-HT2A/C receptors in the rat basolateral amygdala (BLA) inhibits behaviors related to reward-seeking by suppressing principal neuron activity, i.e., neurons that project out of the BLA. These data provide new insight into the role of the BLA in modulating reward-related behaviors. Kallupi et al. (2014) investigated a novel nonpeptidergic Nociception/Orphanin FQ (NOP) receptor agonist that interacted with the GABAergic system in the rat central amygdala (CeA) in a similar manner to the peptide nociceptin and prevented ethanol-induced augmentation of GABA signaling in the CeA, suggesting that the NOP receptor may represent a useful therapeutic target for the treatment of alcoholism. Pava and Woodward (2014) examined the effects of repeated alcohol exposure on cannabinoid regulation of up-states in slice cultures of the prefrontal cortex (PFC) and found that up-state duration was increased after chronic ethanol and withdrawal. Chronic ethanol and withdrawal also blunted the effects of cannabinoid 1 receptor agonism on up-state amplitude and inhibitory currents in PFC neurons. These data suggest that chronic ethanol and withdrawal compromises the control of PFC activity by the cannabinoid system. The cannabinoid system was also the focus of Palomino et al. (2014), who examined the impact of acute and repeated cocaine exposure on endocannabinoid (eCB) and glutamate signaling in the mouse cerebellum. Their findings indicate that acute cocaine modulates the expression of the eCB and glutamate systems. Repeated cocaine results in normalization of glutamate receptor expression, although sustained changes in eCB are observed. These findings in the cerebellum have particular relevance in the context of behavioral sensitization, a critical component in the addiction process. Furthermore, Blanco-Calvo et al. (2014) evaluated whether the endogenous cannabinoid system affects cocaine-induced alterations in cell proliferation in the adult rat and found that while acute cocaine exposure decreased hippocampal cell proliferation, blockade of the cannabinoid receptors restored proliferative actions and prevented the conditioned locomotion induced by cocaine exposure.

As the neurophysiology of alcohol and drugs of abuse in the brain are explored in more detail, an important area of study has emerged concerning sex differences in how drugs and ethanol interact with various brain systems to produce behavioral effects. Melis et al. (2013) examined sex differences in dopamine neuronal properties and activity of the cannabinoid system in the ventral tegmental area (VTA) in the rat and found that females displayed larger depolarization-induced suppression of inhibition (DSI) than male rats via tonic 2-arachidonoylglycerol signaling. These findings highlight sex-specific differences in VTA endocannabinoid activity that may regulate responses to aversive intrinsic properties to cannabinoids and contribute to differences in cannabinoid consumption. McCall et al. (2013) utilized a mouse model of selective deletion of the neuropeptide Y 2 (Y2) receptor in GABA neurons to examine sex differences in the role of Y2 receptor on anxiety and drinking. Females displayed greater basal anxiety, higher levels of ethanol consumption, and faster fear conditioning than males, and Y2 knockout mice exhibited enhanced depressive-like behavior in the forced swim test. This study extends work on sex differences in ethanol consumption and highlights the importance of Y2R function in GABAergic systems in the expression of depressive-like behavior.

The process of addiction is characterized by patterns of addictive behavior, many of which can be modeled in experimental paradigms using rodents. For example, McClure et al. (2014) presented a new method of isolating individual components of impulsive choice, specifically delay discounting and reward quantity in adolescent rats, and find that differences in timing and delay discounting are not causally related, but instead are more likely influenced by a common factor. As impulsive behavior is closely associated with addiction, this important new method allows for an improved understanding of a complex aspect of addictive behavior. Sommer et al. (2014) used a mouse model of rotarod training to demonstrate phase-and region-specific alterations in dopamine receptor binding and transcription levels (decreased D1 binding in the dorsomedial striatum after early training and a reduction in D2-like binding in the dorsolateral striatum after prolonged training) in the dorsal striatum. These findings have profound implications for the role of striatal dopamine in the “automated” behaviors associated with dependence. In another study, Butler et al. (2014) looked at the relationship between early life stress and ethanol self-administration. Adolescent rats exposed to social isolation exhibited a dysregulated hypothalamic-pituitary-adrenal axis seen in the significant correlation between baseline corticosterone levels and increased anxiety as well as increased ethanol intake. These results illustrate the profound effects of early life stress on anxiety and an increased vulnerability for developing addictive disorders.

The breadth and depth of the studies in this topic illustrate the complex actions of alcohol and drugs of abuse on various neurobiological systems. Together this work represents the most current understanding of how acute and/or chronic exposure to abused substances engages and/or pathologically alters distinct brain circuits. Although much progress has been made in understanding addiction as a disease with biological underpinnings, much work is still required to understand the mosaic of actions that drugs of abuse promote in various brain systems and to facilitate the development of therapeutics that can better serve a significant clinical population that struggles with addiction.

## Conflict of interest statement

The authors declare that the research was conducted in the absence of any commercial or financial relationships that could be construed as a potential conflict of interest.
